# Identification of Patients with Sarcopenia Using Gait Parameters Based on Inertial Sensors

**DOI:** 10.3390/s21051786

**Published:** 2021-03-04

**Authors:** Jeong-Kyun Kim, Myung-Nam Bae, Kang Bok Lee, Sang Gi Hong

**Affiliations:** 1 Department of Computer Software, ICT, University of Science and Technology, Daejeon 34113, Korea; kim.jk@etri.re.kr; 2 Intelligent Convergence Research Laboratory, Electronics and Telecommunications Research Institute, Daejeon 34129, Korea; mnbae@etri.re.kr (M.-N.B.); kblee@etri.re.kr (K.B.L.)

**Keywords:** sarcopenia, gait analysis, gait parameter, XAI, inertial measurement units, smart insole, Shapley Additive explanations

## Abstract

Sarcopenia can cause various senile diseases and is a major factor associated with the quality of life in old age. To diagnose, assess, and monitor muscle loss in daily life, 10 sarcopenia and 10 normal subjects were selected using lean mass index and grip strength, and their gait signals obtained from inertial sensor-based gait devices were analyzed. Given that the inertial sensor can measure the acceleration and angular velocity, it is highly useful in the kinematic analysis of walking. This study detected spatial-temporal parameters used in clinical practice and descriptive statistical parameters for all seven gait phases for detailed analyses. To increase the accuracy of sarcopenia identification, we used Shapley Additive explanations to select important parameters that facilitated high classification accuracy. Support vector machines (SVM), random forest, and multilayer perceptron are classification methods that require traditional feature extraction, whereas deep learning methods use raw data as input to identify sarcopenia. As a result, the input that used the descriptive statistical parameters for the seven gait phases obtained higher accuracy. The knowledge-based gait parameter detection was more accurate in identifying sarcopenia than automatic feature selection using deep learning. The highest accuracy of 95% was achieved using an SVM model with 20 descriptive statistical parameters. Our results indicate that sarcopenia can be monitored with a wearable device in daily life.

## 1. Introduction

The interest in maintaining the daily abilities for a healthy retirement life is increasing owing to the increase of the elderly population and the extended life expectancy. Elderly people are susceptible to sarcopenia that is characterized by decreased muscle mass and muscle function owing to nutritional deficiencies and decreased physical activity. Sarcopenia is the cause of numerous senile decays, such as falls, fractures, physical disabilities, depression, poor quality of life, nursing home admission, and even death [1].

Dual energy X-ray absorptiometry (DEXA) and bioelectrical impedance analysis (BIA) are primarily used as tools for diagnosing patients with sarcopenia. The European working group on sarcopenia in older people (EWGSOP) uses the grip strength and walking speed as additional variables to determine the level of sarcopenia [2]. Existing screening methods cannot be applied without expert help. Therefore, a screening study that can be easily conducted in nonhospital settings is required. Studies on muscle reduction and walking speed are actively being conducted. Interestingly, walking speed has allowed independent predictions of mortality [3]. Therefore, gait analysis can be a useful tool to determine muscle loss [4]. Cameras and force plates are gold-standard tools for clinical gait evaluations. Camera-based gait analyses are used in large institutions and university hospitals. In order to accurately analyze gait, it is necessary to capture three-dimensional (3D) motion. Since two-dimensional (2D) cameras cannot detect 3D motion, 2D camera recordings from various angles are needed to visualize and quantify gait [5]. Recently, spatial issues have been solved because 3D data have been easily obtained owing to the development of depth cameras [6]. However, expensive high-speed cameras are required to accurately analyze human gait. Force-plate-based methods [7] are very good tools used to measure the reaction force of gait, and to detect step lengths and widths. However, they have the disadvantage of not acquiring kinematic information.

Inertial measurement units (IMUs) are attracting increased scientific attention as gait analysis tools because the gold-standard tools are difficult to use to conduct gait monitoring at home or outdoors [8,9].

The gait parameters are detected to determine the effective gait condition. The parameters used in clinical analysis are (a) spatial-temporal parameters (e.g., step length, stance phase, swing phase, single support, double support, step time, cadence, and speed) and (b) kinematic parameters (the rotational angles of the sagittal, coronal, and transverse sections of the pelvis, hip, knee, and ankle) [10].

The inertial sensor-based gait parameter detection method derives spatial-temporal parameters from the values of acceleration and angular velocity signals measured by the inertial sensor, and extracts features of descriptive statistics (maximum, mean, standard deviation, etc.) of the acceleration and angular velocity signal. Segmentation of the gait phase is necessary to classify daily activities or to assess pathological gait.

Machine learning algorithms are used as a method for screening various diseases using gait. As a method of detecting gait features, support vector machines (SVM) and random forest (RF) obtained the best results, and high-screening results were obtained using deep learning technology that does not extract features [11]. In the field of gait analysis, domain knowledge to detect gait parameters remains important for designing the inputs of the model. The explainable artificial intelligence (XAI) method is receiving increased attention as a method used to obtain domain knowledge based on machine learning [12].

The aim of this study is to detect parameters from the gait signal measured in the inertial sensors as a screening method for the sarcopenia group and identify the optimal classification method. The highlights and contributions of our work can be summarized as follows: DEXA and grip strength were measured to classify 10 sarcopenia patients and 10 normal volunteers, and inertial gait data were obtained from each participant. Gait parameters effective for identifying sarcopenia were detected using the XAI technique. The optimum classification method was achieved with the use of the various parameters as input.

For gait parameters, the criteria for data analysis were selected to be right because 13 subjects were right-leg dominant and 7 were left-leg dominant. Owing to insufficient data, the effect of the dominant leg could not be analyzed and was, therefore, not considered. Further, the proportion of dominant leg per group (control = 7, sarcopenia = 6) was similar.

The remainder of this paper is organized as follows. Section 2 introduces related studies on detecting gait parameters and classifying diseases. Section 3 presents detailed screening methods for sarcopenia groups, sensor devices, and proposed algorithms. Section 4 presents the results for detection of gait parameters and identification of sarcopenia. Section 5 presents the discussion. Conclusions are listed in Section 6.

## 2. Related Work

### 2.1. Gait Phases

**Figure 1:**
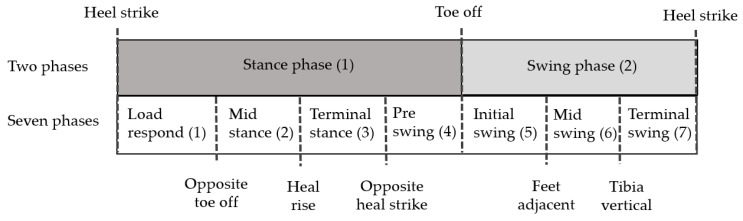
Two and seven gait phases.

Gait describes human walking that exhibits periodic patterns termed as gait cycles. For gait analysis, it is important to detect various gait parameters. The gait parameters are detected based on the gait cycle, and the gait phase is clinically divided into 2–7 phases [13]. Gait is divided into the stance and swing phases; the stance phase refers to the case in which the foot is attached to the ground, and the swing phase refers to the case in which the foot is separated from the ground. The stance and the swing phases are further divided into 2–4 subphases. Whittle et al. [14] divided gait into seven phases. The stance phase was classified into the loading response, mid stance, terminal stance, and preswing phases, and the swing stage was classified into the initial swing, mid swing, and terminal swing phases, as shown in Figure 1.

Heel strike (HS) and toe-off (TO) detection is required to separate the stance and swing phases. Spatial-temporal parameters, such as cadence, stance phase (time), swing phase (time), single-support phase (time), double-support phase (time), step time, and stride time can be detected by mathematical calculations with HS, TO, opposite HS, and opposite TO. Therefore, detection of HS and TO is very important for detecting gait parameters.

Misu et al. [15] detected HS as an acceleration signal and TO based on angular velocity signals. Mo and Chow [16] detected HS and TO based only on residual acceleration. HS yielded the highest acceleration peak, and TO was selected as the phase associated with 2 g or more. Khandelwal et al. [17] detected HS and TO using complex acceleration signals according to continuous wavelet transform (CWT)-based frequency analysis. We obtained very good accuracy by detecting acceleration along the x-axis for HS and acceleration along the z-axis for TO instead of complex acceleration signals through CWT [18]. Based on IMUs attached on the foot, existing work involve 3–4 phases, including HS and TO. More studies have been conducted on the classification of stance rather than the swing phase. The front and rear of the flat foot where the foot touched the ground were classified, and an algorithm was proposed based on the rule and hidden Markov models (HMM). The existing gait phase studies are frequently divided into the HS-FF (TS)-HO-TO phases, as shown in Table 1. The abbreviations for each phase or event are as follows: ST: Stance, SW: Swing, MSw: Mid swing, MSt: Mid stance, HO: Heel off, TS: Toe strike, FF: Flat foot, oHS: Opposite heal strike, oTO: Opposite toe off, HR: Heel rise, FA: Feet adjacent, and TV: Tibia vertical. These are mainly detected using gyroscope data, and the position of the sensor changes from forefoot to hindfoot. Pérez-Ibarra et al. [19] identified HS as the zero-crossing of sagittal angular velocity during the swing phase. TO was determined by the zero-crossing of sagittal angular velocity during negative to positive changes before HS. MSt (from TS to HO) was detected when the resultant angular velocity was below the threshold. Zhao et al. [20] detected HS-FF-HO-TO using inertial sensors and multisensor fusion. MSw and MSt events were detected as the events associated with the minimum magnitudes of specific forces compared with Vicon data. FF and HO were detected when the sagittal angular velocity was almost zero. HS was detected as the zero crossing in the same manner as Pérez-Ibarra’s method, and TO was detected as the maximum value of the sagittal angular velocity. Two negative peaks occurred after the positive signal of the sagittal angular velocity and are related to TO and HS events. The gait phase detection methods in the studies [21,22,23,24,25,26,27,28] listed in Table 1 have been reported by Pérez-Ibarra et al. [19] and Zhao et al. [20].

### 2.2. Patient Identification Using IMU

Studies conducted to identify patients based on inertial sensors include Faller, Parkinson’s disease (PD), and total hip arthroplasty (TPA). Moreover, algorithms such as naive Bayes (NB), SVM, RF, decision tree (DT), k-nearest neighbor (kNN), and deep learning (DL), were applied as classification methods. The spatial-temporal and descriptive statistical parameters derived from the acceleration and angular velocity signals were used as input to the algorithm. Existing studies include the spatial-temporal, descriptive statistics, and frequency parameters. Spatial-temporal parameters are cadence, stride time, stride length, speed, stance, swing, and double and single support. Descriptive statistical parameters include range of motions (ROM), maximum, mean, and standard deviation. Frequency parameters, such as the spectral entropy, median frequency, and fast Fourier transform were used.

Teufl et al. [29] classified TPA patients using stride length, stride time, cadence, speed, hip, and pelvis ROM as features of the SVM, and obtained an accuracy of 97%. Caramia et al. [30] classified PD using the linear discriminant analysis (LDA), NB, k-NN, SVM, SVM radial basis function (RBF), DT, and the majority of votes. The performance of the machine learning technique—the SVM with nonlinear kernel basis—was the best. Howcroft et al. [31] predicted the risk of falls using accelerometer data and used temporal (cadence and stride time) and descriptive statistics (maximum, mean, and standard deviation of acceleration). NB, SVM, and artificial neural networks (ANN) were used as classification methods, and the best single-sensor model was the neural network. Deep learning is currently receiving tremendous attention as a classification algorithm. Additionally, Eskofier et al. [32] classified PD based on the application of AdaBoost, PART, kNN, SVM, and convolutional neural networks (CNN), and CNN yielded the highest accuracy. Deep learning has an advantage in that it can detect features within the algorithm from the raw signal, but Tunca et al. [33] achieved a higher accuracy in long short-term memory (LSTM) when parameters (e.g., speed, stride length, cycle time, stance time, swing time, clearance, stance ratio, and cadence) were used as input compared with raw signals. Zhou et al. [34] classified age groups by dynamic gait outcomes with SVM, RF, and ANN. Gait outcomes that significantly contributed to classification included the root-mean square, cross entropy, Lyapunov exponent, step regularity, and gait speed. In recent years, interest in XAI has been increasing owing to its classification accuracy and to features that significantly contributed to classification. Dindorf et al. [12] used the local interpretable model-agnostic explanations (LIME) to understand the features for identifying total hip arthroplasty (THA), and found that the sagittal movement of the hip, knee, and pelvis as well as transversal movement of the ankle were especially important for this specific classification task, as shown in Table 2.

## 3. Methods

In this section, we present detailed screening methods for sarcopenia groups, sensor devices, and proposed algorithms.

### 3.1. Subject, Equipment, and Data Collection

Ethical approval was obtained from the Chungnam National University Hospital Institutional Review Boards before the study was conducted (File No: CNUH 2019-06-042). We collected gait data from 10 elderly women with sarcopenia and 10 normal women with non-sarcopenia. The diagnosis of the sarcopenia group was selected as the lean mass index (appendicular skeletal muscle mass in kg/height in m^2^) of less than 5.4 kg/m^2^ as a result of DEXA, while the grasp power was less than 18 kg. Population statistics can be found in Table 3. The walking data of the 20 subjects were obtained with a sensor that was attached to their insoles over four walking cycles at the speed of their choice through a straight corridor of 27 m. A total of 80 walking cycles were acquired. The sampling rate of the inertial sensor was measured at 100 Hz.

The proposed insole system obtained Pearson’s correlation, r > 0.9, for HS, TO, opposite HS, and opposite TO, compared to the camera- and force-plate-based clinical standard system. An intraclass correlation >0.9 was obtained based on four measurements. The results achieved the same high validity and reliability as existing inertial system [35,36,37].

The clinical system consisted of 10 cameras (Vicon, Oxford Metrics, Oxford, UK) and four force plates (ATMI, Advanced Mechanical Technology, Watertown, MA, USA). Data analysis was performed with Vicon Polygon 3.5.2 (3.5.2, Oxford Metrics, Oxford, UK). 

The size of inertial insole device was 17 × 25 × 3 mm; the processor was a Nordic nRF52840 (ARM Cortex-M4 32-bit processor with FPU, 64 MHz, Cambridge, UK), the inertial sensor was an Invensense MPU-9250 with 16-bit ADCs, the flash was 512 Mbits, memory was 1 MB flash and 256 kB RAM, and the device supported Bluetooth low energy (BLE) mode.

### 3.2. Extraction of Gait Parameters

The 3-axis acceleration and angular velocity signals of the right foot and the left foot were obtained from the inertial sensor, and spatial-temporal and descriptive statistics parameters were obtained from the signals. The definitions of the gait parameters used in this study are shown in Table 4. The value of the descriptive statistics is the 3-axis inertial signal divided by the gait phases given in Table 4.

To detect the spatial-temporal parameters, the HS, TO, opposite HS, and opposite TO were detected. Acceleration increased rapidly when the swing phase was changed in the stance phase but decreased gradually during the swing phase, and the acceleration value was suddenly zero at HS. The time to return to zero near the minimum value of acceleration ranged from 0.01–0.02 s and was detected at the HS. Given that the x-axis acceleration signal has a minimum value before the stance phase, the minimum value of the acceleration is detected within the interval, and the highest value of the derivative x-axis acceleration (change in acceleration) is then selected as the HS within 10 samples (the minimum value of the acceleration along the x-axis).

TO detection occurred when a rapid rotational force about the y-axis was generated so that the foot came off the ground during gait phases. This constituted a change associated with the swing phase from the stance phase. The gyro sensor in the insole detected an increase when the heel came off the ground and had the highest torque because the greatest force was applied to the foot at TO. Therefore, the maximum value of the gyro y-axis signal was used to identify TO.

To extract the stride and step lengths, we applied a distance estimation algorithm based on zero velocity detection (zero-velocity update) with an extended Kalman filter [38,39].

### 3.3. Extraction of Gait Phase

To divide the gait into seven phases, heel rise (HR), feet adjacent (FA), and tibia vertical (TV) were also detected. 

The HR distinguishes the transition from the mid stance to the terminal stance and depends on the individual and walking speed. In the mid stance phase, the angular velocity of the gyro y-axis (pitch) is close to zero, and then increases to a value (in the counterclockwise direction) as the heel falls off the floor. The mid stance appears at 32% of the walking cycle, and the position where the y-axis angular velocity changes to 0.25 or more was selected as the HR.

The FA values are in positions that separate the initial swing and the mid swing when both feet cross the stance leg on the opposite side of the swing leg in the sagittal plane. The FA occurs in 77% of the gait cycle. When both feet are adjacent, the body is in the highest position and the toes are located closest to the ground [14]. In the case of the inertial sensor attached to the insole, it is difficult to detect the exact point adjacent to the feet. However, assuming that the foot moves the pendulum, the acceleration has a value of zero at the lowest point. Therefore, the FA was detected as the point where the x-axis acceleration became zero.

The TV corresponds to 86% of the gait cycle based on the division of the mid swing and the terminal swing. The TV is a position between the dorsiflexion and the plantarflexion before the subsequent HS [14]. Given that the joint angle is located immediately at zero, the point where the joint angle is zero is defined as the TV. The joint angle is calculated by integrating the angular velocity along the y-axis. However, the error is minimized by using the minimum integration because the cumulative error occurs in obtaining the angle based on integration. At the point at which the joint angle is zero, the x-axis speed is close to zero. In the case of the detection method, when the foot rises to the maximum point after TO in the swing phase, the x-axis speed becomes zero, and the angular rotation speed along the y-axis also has a value of zero. The x-axis acceleration is integrated to obtain the velocity from the point at which the y-axis angular velocity is zero. Accordingly, the point at which the velocity becomes zero is detected by the TV.

### 3.4. Feature Selection

The detected gait parameters differ in their capacity to identify sarcopenia. Applying many dimensional parameters to the classifier can yield poor results. Therefore, it is necessary to reduce the dimensions or select features and apply them to the classifier. In recent years, XAI technology has been attracting attention as a method used to help understand the classification result rather than simply reduce the dimension. XAI presents predictive results for machine learning in a way that humans can understand [40]. Machine learning models based on trees are the most popular nonlinear models in use today [41]. Extreme gradient boosting (Xgboost) proposed by Chen and Guestrin [42] is one of the decision tree ensemble models. Xgboost is an algorithm that improves the performance of the gradient boosting machine (GBM) in terms of speed. The boosting model has a low-overfitting risk because it generates a powerful classifier by updating the parameters of the former classifier iteratively to decrease the gradient of the loss function [43]. By focusing on the model performance, Xgboost has become more complex and lost its interpretability. These models provide an inconsistent measure depending on the tree structure. It only shows the overall importance and not the effect of independent variables. Shapley Additive explanation (SHAP) values are utilized with the intention to improve these problems. The SHAP is a method used to interpret results from tree-based models. The values are based on Shapley values from game theory. The main advantages of the SHAP method are the local explanation and consistency in global model structure. The SHAP value is a numerical expression of how much each feature contributed to creating the total outcome. The contribution of each feature can be expressed as the degree of change in the total outcome when the contribution of that feature is excluded. Equation (1) represents the SHAP value, where $\emptyset_{i}$ is the SHAP value for the data, *n* is the number of feature, *N* is the set all n features, *S* is all features except the *i*-th feature, v(S) is the contribution to the result without the *i*-th feature, and $v\left(S\cup^{\;}\left\{i\right\}\right)$ is the contribution of all features including the *i*-th feature. The degree of contribution of the *i*-th feature is the value obtained by subtracting the sum of the contributions excluding the *i*-th feature from the total contribution.

(1) $$\emptyset_{i}\left(v\right)=\underset{S\epsilon N\backslash \left\{i\right\}}{\sum }\frac{\left|S\right|!\left(n-\left|S\right|-1\right)!}{n!}\left(v\left(S\cup^{\;}\left\{i\right\}\right)-v\left(S\right)\right)$$

**Figure 2:**
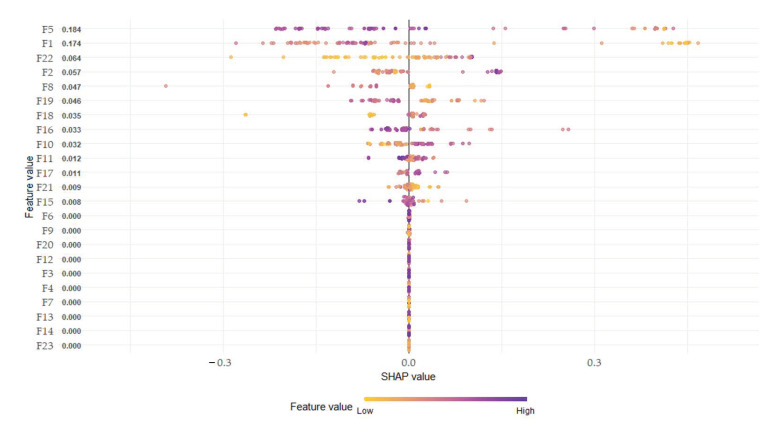
Summary plot of spatial-temporal parameters.

Analysis of summary plot obtained by SHAP can provide the distribution of the impact of each feature. The summary plot superimposes feature importance and feature effects. Each point in the summary plot representsthe SHAP value and observation value for the feature (gait parameters), where the x-axis represents the SHAP value from the scale of negative factors to the scale of positive factors for sarcopenia identification, and the y-axis represents the feature. The features are ordered according to their importance. The color represents the feature value from low (yellow) to high (purple). Therefore, the summary plot shows the magnitude of the positive or negative impact of the feature on the identification of sarcopenia when the value of each parameters is high or low. The SHAP summary plot can be seen in Figure 2 The parameter settings for the SHAP for Xgboost are as follows: Objective: Binary logistic, nroonds: 20, max_depth: 15, gamma: 0.009, and subsample: 0.98. The SHAP for Xgboost are implemented as an R package and is available from Comprehensive R Archive Network (CRAN).

### 3.5. Machine Learning

We explored the most suitable method for identifying sarcopenia using inertial signals and gait parameters. RF, SVM, and multilayer perceptron (MLP) are the most popular machine learning methods in gait analysis and use feature parameters as input. Deep learning models that do not require feature extraction, such as the CNN and LSTM, have yielded the best results in various fields. RF is a decision tree ensemble classifier that combines multiple single classifiers to finalize the results from each classification model through a majority vote or a weighted average. The RF was constructed based on the decision tree so it can classify various categories. It has a fast learning speed and big data processing ability [44]. The number of RF trees was 50, and the number of features was selected to be the square root of the gait parameter; the max depth of trees was 30, and the minimum leaf size of the sample was 1.

SVM is a binary classifier that aims to find the optimal separation hyperplane that maximizes the margin between the two classes. Kernel functions are used to map data to a higher dimensional space, so SVM can compute nonlinear decision boundaries [45]. We explored the linear and RBF kernels, and the parameters were gamma = 1.0 and C = 5.0.

The MLP [46] is a feed-forward neural network with input, hidden, and output layers. The hidden layer employs activation functions to be able to capture nonlinear associations. This model is used for the classification based on feature inputs, unlike the deep learning method. The hyperbolic tangent sigmoidal function (tanh) is used as the activation function in the hidden unit. The scaled conjugate gradient backpropagation algorithm is used to train the network. We chose to use the MLP with two hidden layers that contained 20 hidden units and 70 epochs.

The CNN [47] is composed of one or more convolutional, pooling, and fully connected (FC) layers. In the convolutional layer, the kernel extracts features while traversing the input data at regular intervals. The output of this layer is the feature map. Distinct from the standard ANN, CNN just needs to train the kernels of each convolutional layer. The convolutional operation acts as a feature extractor by learning from the diverse input signals. The extracted features can be used for classification in subsequent layers. In the pooling layer, this is a down-sampling layer. The samples of the most representative features are extracted from the convolutional layer. The sampling method includes the max and average pooling, which is performed by extracting the maximum or the average value of each interval.

In the case of CNN, the raw data of the acceleration and angular velocity are used as input data, and the length of the data is selected to include 100 samples. The CNN architecture was initially implemented with a one-dimensional (1D) convolutional layer with 64 filters, 1 stride, and a kernel size equal to 8. The next layer was a max-pooling layer with a pooling size of 4 and with 4 strides. The third layer was the FC layer with CNN feature inputs and 2014 neuronal outputs. The last layer was another FC layer with neuronal outputs classes and a softmax function.

The recurrent neural network (RNN) [48] architecture was a highly preferred architecture for sequential data. This architecture has been successfully applied to many problems, such as natural language processing, speech recognition, prediction of stock market, and machine translation. Unlike a traditional neural network, the RNN has a learning structure in which all inputs and outputs are connected to each other, so it can memorize previous data and be recursively used as input in the current state. This recurrent connection structure was developed in 1982 by Hopfield [49]. The LSTM network by Hochreiter and Schmidhuber [50] emerged in sequential data analysis as the most extensively used type of RNN architecture. The LSTM has the disadvantage that the input is heavily influenced by the previous input because the input value is time dependent. To eliminate this disadvantage, Bidirectional LSTM (BiLSTM) [51] has been proposed. BiLSTM have forward and backward hidden layers, which are not connected to each other. Thus, they can learn both the prior and subsequent information. The BiLSTM architecture started with two BiLSTM layers with 64 and 32 filters. The layers after BiLSTM were the same as the CNN architectures. The learning rate was 0.0005, and the dropout was 0.2.

The parameters of SVM, RF, MLP, CNN, and BiLSTM were obtained by grid search. Optimal parameters were obtained for various input features; spatial temporal parameters, descriptive statistical parameters for two phases and seven phases, and the parameters that did not affect the results and classification accuracy were selected.

### 3.6. Proposed Data Pipeline

**Figure 3:**
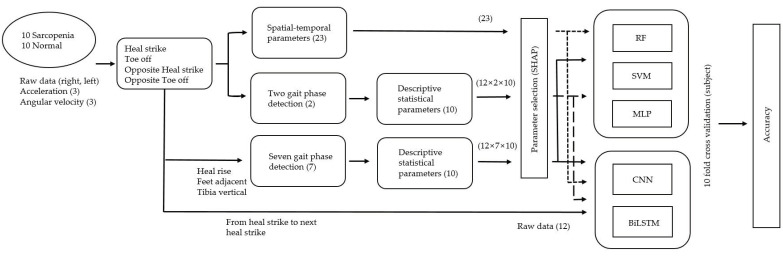
Data processing pipeline for the identification of sarcopenia.

The acceleration and angular velocity walking signals (12-axis) of 20 subjects were detected by using the inertial sensors of both feet. HS, TO, opposite HS, and opposite TO were detected from the inertial signals. Spatial-temporal parameters (23) were calculated using the detected HS and TO of both feet, and HR, FA, and TA were additionally detected. The gait phases were classified into either two or seven phases. The two phases were classified into HS and TO, and the seven phases were classified into HS, opposite TO, HR, opposite HS, TO, FA, and TA. Descriptive statistical parameters (10) were detected for each inertial signal that was divided into two and seven phases. For descriptive statistical parameters, 240 (12 × 2 × 10) parameters and 840 (12 × 7 × 10) parameters were detected for the two and seven phases, respectively, as shown in Figure 3. Of the detected parameters, only 50 were applied to the SHAP in the order of the lowest *p*-value resulting from the independent *t*-test because the descriptive statistical parameters had too many features compared with the data used to apply the SHAP. The 50 parameters were determined using a grid search and the designer’s intuition, and this number did not generate big errors in the results. Parameters 1–20, were used as inputs to RF, SVM, MLP, CNN, and BiLSTM because the SHAP values were 0.002 or more within the top 20. Raw inertial signals from the current to the next HS (one stride) were transformed into 100 samples by spline interpolation because the number of samples for each stride was different, and the samples were used as input for deep learning. For evaluation, nine subjects in each group were used as training data, and one subject in each group was used as test data. Evaluation results averaged the accuracy of 10 evaluations.

## 4. Results

### 4.1. Gait Parameters

The detected spatial-temporal parameters and descriptive statistics parameters for two and seven phases are outlined in the Appendix A. Since the proposed parameter accuracy is calculated based on HS, opposite TO, HR, opposite HS, TO, FA, and TA, these seven events should be accurately detected. HS and TO from the inertial sensor were detected with an error of less than 0.03 s (3 samples) compared to the standard system. HR, FA, and TA were calculated according to the proposed method from the detected HS and TO, and all were detected without error. The results of the application of the SHAP with Xgboost to the spatial-temporal and descriptive statistical parameters for two and seven phases are as follows:

- Spatial-temporal parameter (top 20): 5, 1, 22, 2, 8, 19, 18, 16, 10, 11, 17, 21, 15, 6, 9, 20, 12, 3, 4, and 7.
- Descriptive statistical parameters for two phases (top 20): 52, 126, 37, 97, 8, 51, 144, 211, 24, 3, 232, 115, 116, 31, 57, 50, 109, 43, 100, and 4.
- Descriptive statistical parameters for seven phases (top 20): 196, 524, 504, 97, 3, 231, 526, 507, 430, 187, 380, 8, 130, 57, 51, 200, 828, 283, 523, and 9.

Regarding the spatial-temporal parameters, the parameter for phase (%) was detected as the most important parameter, and time dRL representing the balance of both sides obtained high importance.

In the case of descriptive statistical parameters for two phases, the stance parameters gained higher importance with 13 stance parameters and 7 swing parameters among the top 20 important parameters. Regarding the parameter importance according to the sensor type, the parameters of the gyro sensor were more important with 14 gyroscope sensors and 6 acceleration sensors. The high importance parameters for seven phases included 17 stance parameters and 3 swing parameters, and the stance parameters had the same high importance as those of the two-phase case. In the stance phases, seven mid stance parameters and six loading response parameters gained high importance. In the case of the sensor type, the acceleration x-axis was six parameters, the gyroscope y-axis was five parameters, and the parameter for the direction of the walking was highly important.

### 4.2. Identification of Sarcopenia

Twenty-three spatial-temporal parameters, 240 two-phase descriptive statistical parameters, and 840 seven-phase descriptive statistical parameters were applied as input to the SVM, RF, MLP, CNN, and BiLSTM. In addition, the important parameters of the top 20 of the two- and seven-phase descriptive statistical parameters were applied to each classification algorithm, as shown in Table 5 and Table 6. The application of the spatial-temporal parameters yielded the best results in the MLP. When descriptive statistics were used, sevens phases, which had more information than two phases, yielded outcomes with good accuracy. The highest accuracy was obtained when the parameters detected by the SHAP with the highest importance (ranked from 1 to 20) were used in conjunction with the SVM. The results of using the raw signal, spatial-temporal parameters, and descriptive statistics parameters for the top 20 importance parameters as inputs for deep learning were better when gait parameters were used than when the raw signal was used.

## 5. Discussion

To identify sarcopenia, existing studies involving the sarcopenia and normal groups reported a decrease in walking speed [2,4] and a poor body balance [52]. In gait analysis using inertial sensors, spatial-temporal parameters have traditionally been used as features to conveniently identify diseases such as Faller, PD, and TPA in daily life. In this study, 23 spatial-temporal parameters used in existing disease recognition were detected to identify sarcopenia patients. As a result of detecting the importance of 23 parameters, the top five were found to be single support phase right, stance phase right, stance time dRL, stance phase left, and stance time right. As shown in Figure 2, the SHAP summary plot of spatial-temporal parameters, the single support phase right decreased in the sarcopenia group compared with the normal group and the negative effect (decreased probability of classification as sarcopenia) increased when the single support phase increased. The decrease in the stance phase right and increase in the stance phase left increased the positive effect of the SHAP value. The mean value of sarcopenia group was higher than that of the normal group, but the opposite result of the two legs in the SHAP influence indicated the imbalance of the two leg abilities in sarcopenia patients. When the speed decreased, the single support phase decreased. Therefore, the same results were obtained as the results of previous studies, i.e., muscle reduction decreased the walking speed. The stance time dRL is the difference between the stance time of both feet and has a high value when there is a large difference, indicating poor body balance. When the stance time dRL increased, the effect of SHAP value also increased. The spatial-temporal parameters are good for understanding the gait characteristics of sarcopenia; however, they do not yield sufficient accuracy when used to identify sarcopenia. Therefore, to detect the acceleration and gyroscope 3-axis signal characteristics obtained from the inertial sensor, descriptive statistical parameters of the signal were extracted and used as inputs of the classifier. The gait signal contained unique characteristics for each gait phase. As a result of subdividing the gait phases, detecting descriptive statistical parameters, and applying them to a classifier, a better identification result was obtained in seven phases compared with two phases. There were 12 gait signals from the three-axis acceleration and gyroscope sensor of both feet, and 10 descriptive statistical parameters of each signal were detected. Increasing gait phases also created many parameters that are meaningless in identifying sarcopenia. Among the detected parameters, high accuracy was obtained by detecting an important parameter using the SHAP for identifying sarcopenia. The highest recognition accuracy was obtained when the seven-phase descriptive statistical parameters were used as input to the SVM, RF, and MLP, which were used as inputs for feature selection using domain knowledge. When the top 20 parameters were used, the highest result was obtained in SVM, which yielded the highest performance in binary classification. This can explain why SVM was frequently used in other disease classifications.

Additionally, raw gait signals and gait parameters were used as inputs for the CNN and BiLSTM; however, the accuracy of the identification of sarcopenia and normal group was lower than that of conventional classification methods. To compare the performance of the model, we applied the identification of 20 subjects and obtained an accuracy of 97% in the CNN. There are studies that have shown good performances using deep learning, but exhibited better performances when parameters were used as inputs compared with raw signals. We confirmed that better performance can be obtained when important parameters are used for sarcopenia recognition using XAI rather than traditional deep learning models.

## 6. Conclusions

Based on various classification algorithms, sarcopenia patients were identified by inputting signals from inertial sensors and gait parameters. The spatial-temporal parameters used in the existing clinical evaluation and diagnosis represent a good tool for understanding gait. However, this does not include the features of kinematic signals during the gait cycle. Therefore, the use of descriptive statistical parameters for each gait phase can yield higher accuracy. High performance can be obtained by selecting important descriptive statistical parameters because the use of many parameters as inputs leads to overfitting or to an excessive learning time. Recently, the SHAP received tremendous attention as a feature selection method. Unlike the conventional feature selection method, which selects features with high accuracy, the SHAP has the advantage of lowering the importance of parameters if similar features exist among parameters with high importance. The input that applied the SHAP to the descriptive statistical parameters of sevens phases yielded the best performance. Specifically, it was shown that the signal of the inertial sensor contained abundant information on gait. Therefore, it is possible to diagnose and manage sarcopenia in daily life with a smart insole and not with an expensive clinical tool. Deep learning did not extract effective features from inertial signals. However, large amounts of data and the selection of different deep learning models and parameters can yield good results. Therefore, additional research on deep learning methods used for the identification of sarcopenia using inertial sensors is needed. We will apply various deep learning techniques and deep learning-based XAI techniques in future research to understand the inertial signals of sarcopenia patients. Further, analysis using deep learning requires a large amount of data; therefore, additional clinical evaluations will be conducted to obtain and analyze data of sarcopenia patients by age and dominant leg.

## Figures and Tables

**Table 1:** Existing studies on gait phase detection.

First Author, Year	Phase/Events	Signals	Algorithm Class	Position
Seel et al., 2016 [21]	4/HS-ST-HO-SW	3 Acc + 3 Gyro	Rule-based	Forefoot
Gouwanda et al., 2016 [22]	3/HS-HO-MSw	1 Gyro	Rule-based	Forefoot
Rueterbories et al., 2014 [23]	4/HS-FF-HO-TO	2 Acc	Rule-based	Forefoot
Mariani et al., 2013 [24]	4/HS-TS-HO-TO	3 Acc + 3 Gyro	Rule-based	Forefoot
Sabatini et al., 2005 [25]	4/HS-ST-HO-SW	1 Gyro	Rule-based	Forefoot
Kang et al., 2012 [26]	4/HS-FF-HO-TO	1 Gyro	Rule-based	Forefoot
Mannini et al., 2014 [27]	4/HS-FF-HO-TO	3 Gyro	HMM-based	Forefoot
Abaid et al., 2013 [28]	4/HS-FF-HO-TO	3 Gyro	HMM-based	Forefoot
Zhao et al., 2019 [20]	4/HS-FF-HO-TO	3 Acc + 3 Gyro	HMM-based	Hindfoot
Pérez-Ibarra et al., 2019 [19]	4/HS-TS-HO-TO	1 Gyro	Rule-based	Hindfoot
Ours	7/HS-oTO-HR-oHS-TO-FA-TV	1 Acc + 1 Gyro	Rule-based	Hindfoot

**Table 2:** Existing studies on disease identification using gait parameters.

First Author, Year	Phase/Events	Signals	Position	Classification	Accuracy
Teufl et al., 2019 [29]	Stride length, stride time, cadence, speed, ROM (hip, pelvis)	THA	Hip, thigh, shank, foot	SVM	97%
Caramia et al., 2018 [30]	Step length, step time, stride time, speed, Rom (hip, knee, ankle)	PD	R&L ankle, knee, hip, chest	LDA, NB, k-NN, SVM, SVM rbf, DT, majority of votes	96.88%
Howcroft et al., 2017 [31]	Cadence, stride time maximum, mean, and standard deviation of acceleration	Faller	Head, pelvis, right left shank	NB, SVM, NN	57%
Eskofier et al., 2016 [32]	Energy, maximum, minimum, mean, variance, skewness, kurtosis, fast Fourier transform	PD	Upper limbs	AdaBoost, PART, kNN, SVM, CNN	90.9%
Tunca et al., 2019 [33]	Stride length, cycle time, stance time, swing time, clearance, stance ratio, cadence, speed	Faller	Both feet	SVM, RF, MLP, HMM, LSTM	94.30%
Zhou et al., 2020 [34]	Root mean square, cross entropy, Lyapunov exponent, step regularity, gait speed	Age groups by dynamic gait outcomes	Trunk	SVM, RF, ANN	90%
Dindorf et al., 2020 [12]	Various parameters	THA	Hip, knee, pelvis, ankle	RF, SVM, SVM rbf, MLP	100%
Ours	Various parameters	Sarcopenia	Both feet	RF, SVM, MLP, CNN, BiLSTM	95%

**Table 3:** Population statistics for normal and sarcopenia groups.

Parameter	Normal	Sarcopenia
Age (years)	69.6 ± 3.0	71.1 ± 2.0
Height (cm)	153.5 ± 5.0	151.0 ± 4.8
Weight (kg)	60.8 ± 5.1	52.7 ± 5.0
Feet size (mm)	238.0 ± 5.1	232.0 ± 5.5
Grasp power right (kg)	22.5 ± 2.6	14.4 ± 3.5
Grasp power left (kg)	22.3 ± 2.8	14.2 ± 3.7
ASM (kg)	14.7 ± 1.6	11.3 ± 0.9
LMI (kg/m^2^)	6.3 ± 0.4	4.9 ± 0.2

**Table 4:** Definition of gait parameters.

Gait Parameters	Definition
Spatial-temporal Parameters	
Cadence	Number of steps acquired per minute
Stance phase (time)	Percent (time) starting with HS and ending with TO of the same foot
Swing phase (time)	Percent (time) starting with TO and ending with HS of the same foot
Single support phase (time)	Percent (time) when only one foot is on the ground
Double support phase (time)	Percent (time) when both feet are on the ground
Step time (length)	Distance by which a foot moves in front of the other foot. The sum of two successive step lengths corresponds to stride length
Stride length	Distance starting with HS and ending with next HS of the same foot
Phase (time) dRL	Absolute values of the difference between the right and left sides of the stance and swing phases (time)
Speed	Stride length/cycle time
Descriptive Statistical Parameters	
Max	Greatest values
Min	Least or smallest values
Standard deviation (STD)	Standard deviation of values
AbSum	Absolute sum of values
Root-mean-square (RMS)	Arithmetic mean of the squares of a set of values
Kurtosis	Assesses whether the tails of a given distribution contain extreme values
Skewness	A measure of the asymmetry of the probability distribution of a real-valued random variable about its mean
MMgr	Gradient from maximum to minimum of values
DMM	Difference between max and min of values
Mdif	Maximum for the difference between two successive values

**Table 5:** Evaluation result of support vector machines (SVM), random forest (RF), and multilayer perceptron (MLP) (accuracy (standard deviation), %).

Parameters	SVM	RF	MLP
Spatial-temporal (23)	65 (21.08)	75 (24.47)	77 (21.1)
Two phases descriptive statistics (2 × 12 × 10)	50 (27.76)	52.5 (24.47)	50 (11.25)
Two phases descriptive statistics (top 20)	80 (22.20)	77.5 (25.81)	77.5 (24.39)
Seven phases descriptive statistics (7 × 12 × 10)	48.75 (3.95)	60 (27.66)	66.3 (17.5)
Seven phases descriptive statistics (top 20)	95 (15.81)	85 (20.28)	90 (20)

**Table 6:** Evaluation result of deep learning models (accuracy (standard deviation), %).

Parameter	CNN	BiLSTM
Raw IMU data (100 × 12)	54.2 (20.72)	45.3 (17.50)
Spatial-temporal (23)	74.76 (24.01)	62.5 (30.33)
Two phases descriptive statistics (top 20)	69.75 (22.53)	56.25 (16.05)
Seven phases descriptive statistics (top 20)	87.5 (19.36)	86.26 (19.72)

## Data Availability

The data are not publicly available due to company security policy and personal protection of subjects. Data are available from the authors upon reasonable request.

## Appendix A

**Table A1:** Spatial-temporal parameters.

Number	Subject	Normal	Sarcopenia	Normal	Sarcopenia	*T*-Test (Mean)	
	Parameter	Mean	Mean	STD	STD	*p*-Value	Statistic
1	Stance phase_right (%)	60.263	60.432	0.683	0.925	0.697	−0.358
2	Stance phase_left (%)	59.870	60.808	0.772	1.042	0.014	−2.250
3	Swing phase_right (%)	39.736	39.567	0.683	0.925	0.697	0.358
4	Swing phase_left (%)	40.129	39.191	0.772	1.042	0.014	2.250
5	Single support phase_right (%)	40.182	39.141	0.926	1.202	0.006	2.486
6	Single support phase_left (%)	39.826	39.633	0.901	1.169	0.657	0.439
7	Double support phase (%)	19.975	21.101	2.149	4.038	0.142	−1.347
8	Stance time_right (s)	0.604	0.615	0.013	0.017	0.323	−0.615
9	Stance time_left (s)	0.600	0.618	0.013	0.017	0.091	−1.250
10	Swing time_right (s) (stride time)	0.415	0.417	0.009	0.012	0.588	0.180
11	Swing time_left (s)	0.418	0.414	0.009	0.012	0.250	1.713
12	Single support_left (s)	0.401	0.391	0.006	0.008	0.013	2.300
13	Double support (s)	0.200	0.210	0.018	0.034	0.186	−1.216
14	Single support_right (s)	0.397	0.395	0.006	0.007	0.642	0.459
15	Stride length (m)	0.954	0.915	0.132	0.148	0.149	1.297
16	Step length_right (m)	0.483	0.467	0.141	0.147	0.226	1.050
17	Step length_left (m)	0.471	0.448	0.123	0.148	0.107	1.482
18	Step time_right (s)	0.515	0.523	0.009	0.011	0.262	−0.697
19	Step time_left (s)	0.501	0.506	0.009	0.011	0.531	−0.114
20	Cadence (steps/min)	123.331	121.978	0	0	0.399	0.251
21	Stance phase dRL	1.224	1.812	0.007	0.011	<0.001	−3.973
22	Stance time dRL	0.014	0.021	1.025	1.405	<0.001	−3.846
23	Swing time dRL	0.012	0.019	0.901	1.319	<0.001	−4.032

**Table A2:** Two phase descriptive statistical parameters.

		Right										Left									
	Parameter	Max	Min	STD	AbSum	RMS	Ku	Ske	MMgr	DMM	Mdif	Max	Min	STD	AbSum	RMS	Ku	Ske	MMgr	DMM	Mdif
Stance phase	AccX	1	2	3	4	5	6	7	8	9	10	121	122	123	124	125	126	127	128	129	130
	AccY	11	12	13	14	15	16	17	18	19	20	131	132	133	134	135	136	137	138	139	140
	AccZ	21	22	23	24	25	26	27	28	29	30	141	142	143	144	145	146	147	148	149	150
	GyroX	31	32	33	34	35	36	37	38	39	40	151	152	153	154	155	156	157	158	159	160
	GyroY	41	42	43	44	45	46	47	48	49	50	161	162	163	164	165	166	167	168	169	170
	GyroZ	51	52	53	54	55	56	57	58	59	60	171	172	173	174	175	176	177	178	179	180
Swing phase	AccX	61	62	63	64	65	66	67	68	69	70	181	182	183	184	185	186	187	188	189	190
	AccY	71	72	73	74	75	76	77	78	79	80	191	192	193	194	195	196	197	198	199	200
	AccZ	81	82	83	84	85	86	87	88	89	90	201	202	203	204	205	206	207	208	209	210
	GyroX	91	92	93	94	95	96	97	98	99	100	211	212	213	214	215	216	217	218	219	220
	GyroY	101	102	103	104	105	106	107	108	109	110	221	222	223	224	225	226	227	228	229	230
	GyroZ	111	112	113	114	115	116	117	118	119	120	231	232	233	234	235	236	237	238	239	240

**Table A3:** Seven phase descriptive statistical parameters.

		Right										Left									
	Parameter	Max	Min	STD	AbSum	RMS	Ku	Ske	MMgr	DMM	Mdif	Max	Min	STD	AbSum	RMS	Ku	Ske	MMgr	DMM	Mdif
Loading response	AccX	1	2	3	4	5	6	7	8	9	10	421	422	423	424	425	426	427	428	429	430
	AccY	11	12	13	14	15	16	17	18	19	20	431	432	433	434	435	436	437	438	439	440
	AccZ	21	22	23	24	25	26	27	28	29	30	441	442	443	444	445	446	447	448	449	450
	GyroX	31	32	33	34	35	36	37	38	39	40	451	452	453	454	455	456	457	458	459	460
	GyroY	41	42	43	44	45	46	47	48	49	50	461	462	463	464	465	466	467	468	469	470
	GyroZ	51	52	53	54	55	56	57	58	59	60	471	472	473	474	475	476	477	478	479	480
Mid stance	AccX	61	62	63	64	65	66	67	68	69	70	481	482	483	484	485	486	487	488	489	490
	AccY	71	72	73	74	75	76	77	78	79	80	491	492	493	494	495	496	497	498	499	500
	AccZ	81	82	83	84	85	86	87	88	89	90	501	502	503	504	505	506	507	508	509	510
	GyroX	91	92	93	94	95	96	97	98	99	100	511	512	513	514	515	516	517	518	519	520
	GyroY	101	102	103	104	105	106	107	108	109	110	521	522	523	524	525	526	527	528	529	530
	GyroZ	111	112	113	114	115	116	117	118	119	120	531	532	533	534	535	536	537	538	539	540
Terminal stance	AccX	121	122	123	124	125	126	127	128	129	130	541	542	543	544	545	546	547	548	549	550
	AccY	131	132	133	134	135	136	137	138	139	140	551	552	553	554	555	556	557	558	559	560
	AccZ	141	142	143	144	145	146	147	148	149	150	561	562	563	564	565	566	567	568	569	570
	GyroX	151	152	153	154	155	156	157	158	159	160	571	572	573	574	575	576	577	578	579	580
	GyroY	161	162	163	164	165	166	167	168	169	170	581	582	583	584	585	586	587	588	589	590
	GyroZ	171	172	173	174	175	176	177	178	179	180	591	592	593	594	595	596	597	598	599	600
Pre swing	AccX	181	182	183	184	185	186	187	188	189	190	601	602	603	604	605	606	607	608	609	610
	AccY	191	192	193	194	195	196	197	198	199	200	611	612	613	614	615	616	617	618	619	620
	AccZ	201	202	203	204	205	206	207	208	209	210	621	622	623	624	625	626	627	628	629	630
	GyroX	211	212	213	214	215	216	217	218	219	220	631	632	633	634	635	636	637	638	639	640
	GyroY	221	222	223	224	225	226	227	228	229	230	641	642	643	644	645	646	647	648	649	650
	GyroZ	231	232	233	234	235	236	237	238	239	240	651	652	653	654	655	656	657	658	659	660
Initial swing	AccX	241	242	243	244	245	246	247	248	249	250	661	662	663	664	665	666	667	668	669	670
	AccY	251	252	253	254	255	256	257	258	259	260	671	672	673	674	675	676	677	678	679	680
	AccZ	261	262	263	264	265	266	267	268	269	270	681	682	683	684	685	686	687	688	689	690
	GyroX	271	272	273	274	275	276	277	278	279	280	691	692	693	694	695	696	697	698	699	700
	GyroY	281	282	283	284	285	286	287	288	289	290	701	702	703	704	705	706	707	708	709	710
	GyroZ	291	292	293	294	295	296	297	298	299	300	711	712	713	714	715	716	717	718	719	720
Mid swing	AccX	301	30	303	304	305	306	307	308	309	310	721	722	723	724	725	726	727	728	729	730
	AccY	311	312	313	314	315	316	317	318	319	320	731	732	733	734	735	736	737	738	739	740
	AccZ	321	322	323	324	325	326	327	328	329	330	741	742	743	744	745	746	747	748	749	750
	GyroX	331	332	333	334	335	336	337	338	339	340	751	752	753	754	755	756	757	758	759	760
	GyroY	341	342	343	344	345	346	347	348	349	350	761	762	763	764	765	766	767	768	769	770
	GyroZ	351	352	353	354	355	356	357	358	359	360	771	772	773	774	775	776	777	778	779	780
Terminal swing	AccX	361	362	363	364	365	366	367	368	369	370	781	782	783	784	785	786	787	788	789	790
	AccY	371	372	373	374	375	376	377	378	379	380	791	792	793	794	795	796	797	798	799	800
	AccZ	381	382	383	384	385	386	387	388	389	390	801	802	803	804	805	806	807	808	809	810
	GyroX	391	392	393	394	395	396	397	398	399	400	811	812	813	814	815	816	817	818	819	820
	GyroY	401	402	403	404	405	406	407	408	409	410	821	822	823	824	825	826	827	828	829	830
	GyroZ	411	412	413	414	415	416	417	418	419	420	831	832	833	834	835	836	837	838	839	840
